# Innovative Interventions: Caring for Persons With Alzheimer’s Disease and Their Caregivers

**DOI:** 10.1093/geroni/igaa057.2801

**Published:** 2020-12-16

**Authors:** Stacy Andersen, Allison Gibson, Katherine Marx

## Abstract

There are 5.8 million Americans living with Alzheimer’s disease and more than 16 million Americans providing unpaid care for people with AD and related dementias. Since a treatment that can slow or stop progression of this disease has yet to be discovered, novel interventions are sorely needed to maintain cognitive function and quality of life among individuals with dementia, improve the health and well-being of caregivers, and provide assistance in caregiving duties. This symposium addresses novel interventions in the dementia care continuum ranging from social and leisure activities for improving cognition to incorporation of emerging technologies to assist with caregiving and provides recommendations and priorities for future studies. The first presentation introduces evidence that participation in an intergenerational choir can improve cognition, social connectedness, and quality of life among people with dementia and their caregivers. The second presentation systematically assesses recent randomized controlled trials of computerized cognitive training (i.e., brain games) aimed at improving cognitive function among individuals with cognitive impairment or dementia. The third presentation examines the evidence that interventions employing artificial intelligence such as robots may improve care for persons with Alzheimer’s disease and caregivers’ quality of life and provides suggestions for future studies to better assess the efficacy of these interventions. The session concludes with a presentation on a survey method used to build consensus among a panel of experts across academia and industry which identified the emerging technologies that are expected to become the most prevalent in dementia care and provides recommendations for limiting associated risks.

